# Involvement of the superior colliculi in crossmodal correspondences

**DOI:** 10.3758/s13414-024-02866-x

**Published:** 2024-02-28

**Authors:** John McEwan, Ada Kritikos, Mick Zeljko

**Affiliations:** School of Psychology, The University of Queensland, St. Lucia, Queensland 4072 Australia

**Keywords:** Multisensory processing, Neural mechanisms, Signal detection theory

## Abstract

There is an increasing body of evidence suggesting that there are low-level perceptual processes involved in crossmodal correspondences. In this study, we investigate the involvement of the superior colliculi in three basic crossmodal correspondences: elevation/pitch, lightness/pitch, and size/pitch. Using a psychophysical design, we modulate visual input to the superior colliculus to test whether the superior colliculus is required for behavioural crossmodal congruency effects to manifest in an unspeeded multisensory discrimination task. In the elevation/pitch task, superior colliculus involvement is required for a behavioural elevation/pitch congruency effect to manifest in the task. In the lightness/pitch and size/pitch task, we observed a behavioural elevation/pitch congruency effect regardless of superior colliculus involvement. These results suggest that the elevation/pitch correspondence may be processed differently to other low-level crossmodal correspondences. The implications of a distributed model of crossmodal correspondence processing in the brain are discussed.

Although humans perceive the world as a singular combined experience, perception is constructed from many concurrent multisensory inputs. To create this coherent experience, these multisensory inputs need to be identified correctly as belonging either together or apart (Ernst & Bülthoff, 2004; King & Calvert, 2001; Noppeney, 2021). This capacity is what allows us to attribute a bird call to a bird, rather than the branch it sits on, and forms part of the bedrock for meaningful holistic perception. The brain combines a variety of methods, or ‘integration cues’ (Stein et al., 2014), to make these judgements of origin. One of the most fundamental integration cues is spatiotemporal coincidence. If multiple sensory signals appear to coincide in location and time, they are more likely to be attributed to the same object or event (Spence, 2007; Starke et al., 2020). In our bird example, the visual signal of the bird aligns with the auditory signal in location and time. Another integration cue is semantic congruence. If the two signals align in their identity or meaning, they are more likely to be integrated than if they do not. Returning to our bird, the observer is likely familiar with bird calls, and draws some semantic association of ‘birdness’ from both the image and sound of the bird (Chen & Spence, 2017; Doehrmann & Naumer, 2008; Spence, 2007). There are obvious benefits to this use of multiple cues. It is possible that one form of cue information is unavailable for instance. If the animal is unfamiliar and nonprototypical, thereby losing semantic information, or if audio-visual signal is worsened by noise, it is evolutionarily adaptive for the brain to draw from a variety of cues to determine multisensory integration. One category of integration cues, the topic of this paper, are crossmodal correspondences.

A crossmodal correspondence (CMC) refers to a consistent association between sensory features from different modalities that are dependent on each individual feature’s magnitude (see Spence, 2011, for a review). When these features veridically match their mental association, they are more likely to integrate than if they do not (Spence, 2011). The lightness/pitch correspondence is a good example of this association. There is ample evidence that when a participant is presented with white or black visual targets, alongside a high- or low-pitched tone, they will preferentially pair white with high pitch and black with low pitch. The association can be observed in accuracy and reaction time benefits for the preferred pairings (white/high pitch, black/low pitch) compared with the unpreferred pairings (white/low pitch, black/high pitch), perceptual disambiguation (such as Rubin face–vase illusion; Zeljko et al., 2021), as well as enhanced integration in tasks like the ventriloquism paradigm (Ben-Artzi & Marks, 1995; Bien et al., 2012; Marks, 1987; Parise & Spence, 2008). Some audio-visual associations include elevation/pitch, lightness/pitch, brightness/loudness, size/pitch, shape/pitch, and motion/pitch (Ben-Artzi & Marks, 1995; Bernstein & Edelstein, 1971; Clark & Brownell, 1976; Gallace & Spence, 2006; Marks, 1987; Melara, 1989). However, evidence exists for at least one CMC involving each of the traditional sensory modalities (Spence, 2011), as well as temperature (Spence, 2020; Wang & Spence, 2017), proprioception (Shinohara et al., 2020), and pain (Pomper et al., 2013).

Researchers have long been interested in whether these CMC associations can be characterized as top-down decisional processes or bottom-up sensory driven processes. A bottom-up process is driven by stimuli attributes while a top-down process is driven by memory, attention, context, and goal-directed processes (McMains & Kastner, 2011). Increasingly, it is becoming apparent that both processes contribute to the effect. One feature of CMCs which suggests top-down processes is that they are dependent on relative stimulus values rather than absolute. This was first demonstrated by Chiou and Rich (2012), who presented participants with irrelevant auditory tones of 100 Hz, 900 Hz, and 1700 Hz alongside a visual target above or below fixation. The elevation/pitch congruency mapping depended on the pair of tones presented. When presented with the 100 Hz and 900 Hz tones, the 100 Hz was matched to low elevation and the 900 Hz to high, but when presented with the 900 Hz and 1700 Hz tones, participants matched the 900 Hz tone to low elevation, and the 1700 Hz to high elevation. This was replicated by Brunetti et al. (2017) using the size/pitch correspondence, who further found that a subject’s relative perception of whether a tone was high or low could be changed on a trial-to-trial basis. This evidence for relative differences suggests an influence of later neural processes on the CMC effect, and is inconsistent with a bottom-up effect which one would expect to depend on absolute stimulus values.

Contrasting this top-down evidence however, CMCs patterns are consistent with bottom-up effects in many paradigms. Using signal detection theory, Mossbridge et al. (2011) found sensitivity (*d*′) for visual discrimination improved with CMC congruency, without a corresponding criterion shift. Typically, the SDT outputs of *d*′ and criterion are thought to reflect sensory and decisional processes, respectively. Zeljko et al. (2019) demonstrated similar findings in the elevation/pitch and lightness/pitch correspondence, additionally ruling out attentional cueing as a possible cause of this sensitivity change. Previously some authors had proposed multisensory cueing of attention from one modality to another, as a top-down cause of CMC effects (Chiou & Rich, 2012). Another way in which CMCs exhibit bottom-up processes is in how they influence responses to ventriloquism tasks. Parise and Spence (2008) showed that CMC congruency enhances multisensory integration by acting as a cue in a temporal order judgment ventriloquism paradigm. Participants were more likely to integrate temporally or spatially offset audio-visual targets if they were congruent. Specifically, they found a significant perceptual benefit in ‘just noticeable difference’ (JND) with congruency, but not ‘point of subjective equality’ (PSE). In ventriloquism tasks and others, JND is a measure of the smallest possible difference in stimuli that a participant requires to discriminate them, while PSE is a measure of maximum participant uncertainty. This lack of PSE effect rules out any appeal to criterion change or response bias in interpreting their results, typically regarded as top-down factors. Parise and Spence (2008) describe this as a ‘genuine perceptual effect.’ These results were replicated in another ventriloquism task, looking at both temporal and spatial ventriloquism, and reporting either temporal order or spatial location (Parise & Spence, 2009). Both tasks again suggested a genuine multisensory binding effect underpinned the ventriloquism effect, rather than a criterion bias. Finally, there is strong evidence that CMCs are processed in an automatic manner, independently of attention (Spence & Deroy, 2013). Many top-down effects require some level of attention to engage higher-level processes. Although the above studies do not rule out all top-down effects, this evidence together suggests that top-down processes are insufficient to explain CMC effects.

Another way to explore the processing of CMCs is in studying the brain regions involved in CMC congruency effects. Many psychological processes can be classified into early or late processes, reflecting how early in the neural pathway the effect manifests. Looking first at EEG, there are conflicting findings. Bien et al. (2012) conducted an EEG/TMS study on the size/pitch correspondence in a spatial ventriloquism task. Participants were presented with large or small visual target, alongside a high- or low-pitched tone. The auditory and visual targets were presented concurrently but spatially offset. Participants were then required to identify the location the sound originated from. Accuracy in this spatial localization was diminished when the auditory target pitch was congruent with the visual target size, suggesting participants’ percept of the auditory target was drawn towards the visual when both were congruent (and away from its veridical position). Using behavioural and EEG results, they traced the origin of this crossmodal mapping to right intraparietal sulcus (IPS) involvement at 250 ms. They then used TMS to inhibit right IPS activity and found the effect of size/pitch congruency on accuracy disappeared. The authors interpret this finding as strongly supporting a late multisensory process behind size/pitch congruency in this paradigm as the IPS is a high order region of multisensory processing.

In contrast to Bien et al.’s (2012) findings, Kovic et al. (2010) conducted an EEG study on sound symbolism of words, pairing rounded sounding letters with rounded visual shapes. Participants were presented with shapes of varying degrees of angularity, from rounded to sharp, accompanied by a nonsense word of predominantly ‘round’ letters (bouba, maluma, etc.) or predominantly ‘sharp’ letters (kiki, takete, etc.). Behaviourally, participants were significantly faster to match to congruent pseudoword image pairs than incongruent (round letters/round shape, spiky letters/spiky shape are congruent pairs, mismatch is incongruent) in a button-press task. In their EEG data, they found significant differences in activation in the occipital lobe (O1 & O2) between congruent and incongruent images as early as 140 ms after presentation. Activity in the occipital lobe at 140 ms post presentation is more consistent with an early sensory process driving this crossmodal effect.

Finally, a study by Sciortino and Kayser (2023) looked for integration of pitch with size, hue, and chromatic saturation in the primary visual cortex using steady state visual evoked potentials (SSVEP). The authors were motivated in part by the previous conflicting findings in CMC EEG data. Their findings showed that the occipital regions of the brain were sensitive to the size/pitch correspondence, and their source analysis suggested the primary visual cortex as the site of origin. Although this does not rule out involvement of the right intraparietal sulcus as Bien et al. (2012) found, this further suggests that there is some involvement of the primary sensory cortices, implicating the involvement of a low-level process. 

The fMRI work with crossmodal correspondences is similarly mixed. McCormick et al. (2018) found congruency differences in inferior frontal and inferior parietal cortex in an elevation/pitch one-back design. Though they concluded this activation most likely reflected late-attentional differences, their behavioural task found no congruency effect on accuracy, raising questions as to whether they truly observed a CMC effect. Contrary to this, Peiffer-Smadja and Cohen (2019) found congruency driven differences in activation of the dorsolateral prefrontal cortex and the auditory cortex when presenting participants with either ‘round’ or ‘sharp’ words, alongside round or sharp visual shapes. They also observed a correlation between the behavioural CMC effect with fMRI activity in the occipitotemporal visual cortex. These findings suggest a nuanced explanation of the effect. Dorsolateral prefrontal cortex activation is consistent with late top-down processing, but auditory and visual cortex activation suggest early processing of these CMC effects. At this stage, it remains plausible that CMC effects manifest at multiple stages in the perceptual process, and throughout a variety of brain structures.

Based on these results, we can speculate about the regions involved in CMC processing. The best explanation, consistent with all the results thus far, is that CMCs represent a combination of both high and low-level processes, and therefore exist as a distributed process across many regions of the brain. This is consistent with the wide variety of reported CMC pairings, from basic visual and auditory pairings like elevation/pitch, through to high complex pairings like music/art (Spence, 2011). Regarding pairings of high complexity, we can point to particular multisensory regions such as the parietal sulcus, and the dorsolateral prefrontal cortex. For low complexity parings, we can propose that there is a direct relay of information between V1 and A1. Some authors such as Zeljko et al. (2019) have speculated that CMC information is communicated directly between primary sensory cortices, but this has yet to be experimentally verified. Nonetheless, all of the previous neuroimaging work to date has only considered these high and low-level cortical routes of information transfer, and so has focused on cortical effects.

An alternative to this sensory pathway is the subcortical sensory system, via the superior colliculus (SC). The SC is a midbrain structure responsible for reflexive orienting in mammals. It does this through a variety of visual, auditory, and tactile inputs, using spatiotemporal coincidence as a cue to integrate crossmodally (Meredith & Stein 1986a, b). It receives these inputs much earlier than the primary sensory cortices. Visual information for instance, is directly sourced from the retina, while auditory information is passed to it from the inferior colliculi before reaching the primary auditory cortex (Jay & Sparks, 1987; May, 2006). It is also known to have a large number of efferent connections to other sensorimotor regions in the brain, including the thalamus, primary visual and auditory cortices, cerebellum, basal ganglia, spinal cord, parietal and frontal lobe eye fields.

The purpose of the current study is to expand on our current understanding of the brain regions involved in crossmodal correspondence by examining whether basic low-level CMCs require the presence of the subcortical pathway to manifest behaviourally. If the CMC congruency effect is unable to manifest when the subcortical pathway is unavailable, this strongly suggests that the superior colliculus is involved in the process. If the CMC effect manifests regardless of subcortical processing of the stimulus, we can infer that the SC is uninvolved, and that audio-visual CMCs are communicated via cortical projections.

To examine the superior colliculus psychophysically, we used a design employed by Leo et al. (2008) and Bertini et al. (2008) that exploits the neuroanatomical characteristics of the SC. The SC is thought to not receive inputs from short-wavelength S cones in the retina (Marrocco & Li, 1977; Sumner et al., 2002), therefore, by constructing visual targets which predominantly activate S cones, one can create stimuli to which the SC is effectively blind. Leo et al. (2008) examined the redundant targets effect, tasking participants with responding as quickly as possible to visual, auditory, or audio-visual targets in which the visual target was either blue (short-wavelength) or red (long-wavelength). While they found a multisensory reaction time benefit for both red and blue targets, only red audio-visual targets exhibited multisensory integration whereas blue audio-visual targets did not, demonstrating the critical role of the SC in integrating visual and auditory information.

Bertini et al. (2008) applied this approach to examine temporo-nasal asymmetry in superior colliculus multisensory integration. Their findings show again that red audio-visual stimuli were integrated while blue audio-visual stimuli were not. Further demonstrating how this method is measuring SC activity, the magnitude of integration in the red condition was greater for stimuli presented in the temporal hemifield than stimuli presented in the nasal hemifield. Anatomical studies of cats suggest that the temporal hemifield has a stronger input to the SC than the nasal hemifield (Sherman, 1974).

It is worth noting that the evidence for this method of isolating S-cone activity to examine the SC is mixed in animal models. Early physiological work such as Marrocco and Li (1977) in anesthetized macaques suggested that the SC received no chromatic information. More recent work has begun to contradict this. White et al. (2009) showed that the SC in awake rhesus monkeys was responsive to colour in the intermediate layers, but not the superficial layers. In addition, the visual onset latency for chromatic information was 30-35ms later than luminance information, suggesting they may not share the same pathway. Tailby et al. (2012) found this did not replicate in anesthetized marmosets however, instead finding that S-cone activity does not contribute to visual responses in the SC. Finally, Hall and Colby (2014) demonstrated evidence that S cones do produce input in the macaque SC, directly contradicting the original studies on macaques such as Marrocco and Li. In summary, animal evidence regarding the use of S cones to isolate SC activity is mixed, but behavioural evidence in humans is fairly strong. The results should be treated as a tentative first investigation of crossmodal correspondence at the level of the superior colliculus.

In the present study, this methodology is used to consider involvement of the superior colliculus in crossmodal correspondences between simple visual and auditory features. We chose to examine three CMCs: elevation/pitch, lightness/pitch, and size/pitch. These CMCs were chosen for two reasons. First, the correspondences are between relatively simple stimulus features. If the superior colliculus does influence CMC effects, the CMC information must be simple enough to be processed there. Second, we wanted some small variation in complexity with each pairing, to allow comparison between CMCs. Zeljko et al. (2019) found evidence suggesting low-level origins of the elevation/pitch and lightness/pitch effects. These features are plausibly processed at the SC level. Size was included as an incrementally more complex visual feature, as it requires multiple cells to encode for size together.

If we can render the SC visually blind in one condition, we can compare behavioural responses with basic CMCs, both with, and without SC involvement. To achieve this, participants completed a visual discrimination where they were presented with a visual target accompanied by a high- or low-pitched tone. The visual target always varied in elevation (above/below), and would further vary in lightness (light/dark) or size (big/small) depending on the relevant CMC. This was completed twice, once with red stimuli and once with blue stimuli. These analyses were done with signal detection theory because Zeljko et al. (2019) and Mossbridge et al. (2011) have previously found sensitivity, but not criterion, changes with CMC congruency. Considering this, we chose to only examine sensitivity (d’) effects in our data. To prevent potential feedback from V1 to the SC, the visual target was forwards and backwards luminance masked. This method of preventing feedback was successfully used by Leo et al. (2008) and Bertini et al. (2008).

First, based on established CMC congruency effects, we predict that when the visual target is red, discrimination sensitivity (*d*′) in each task will be significantly greater when the visual and auditory targets are congruently paired than incongruently paired. Second, and critically for our research question, if the superior colliculus is involved in the relevant crossmodal correspondence, we predict that when the visual target is blue, the aforementioned congruency effects will be absent, and only manifest when the visual target is red. Regarding specific predictions as to whether each CMC requires SC activity, we expect that size/pitch is less likely to require the SC, compared with lightness/pitch and elevation/pitch. As described earlier, the greater complexity of size as a feature makes it less likely that the SC is able to make use of this information when performing MSI.

## Method

### Participants

Thirty-four participants (11 male, 23 female; age = 23.6 years ± 4.4 years) were recruited through the University of Queensland research participation system for the elevation/pitch experiment, while 30 participants (seven male, 23 female; age 24.2 ± 5.5 years) were recruited to complete both the lightness/pitch and size/pitch experiments. Sample size was determined a priori through a power analysis, suggesting that for an effect size of 0.25, alpha of 0.05, and power of 0.8, 24 participants would be required for each experiment. Due to participant removal in data cleaning, we aimed for approximately 30 participants in each experiment. The effect size of 0.25 (Cohen’s *F*) was taken from previous signal detection congruency effects in Zeljko et al. (2019). Participation was voluntary, and participants were compensated either $20 AUD or course credit for a first-year psychology course they were enrolled in. The experiment was approved by the University of Queensland’s School of Psychology ethical review process.

### Design

All three experiments follow a 2 (visual feature) × 2 (pitch) × 2 (colour) within-groups factorial design, where participants are required to make an unspeeded discrimination about the visual feature. The visual feature to be discriminated was elevation in Experiment 1 (above versus below fixation), lightness in Experiment 2 (light versus dark), and size in Experiment 3 (small versus big). The auditory feature was a pure tone (high or low pitch) in all experiments. The colour was red or blue. The colour was blocked while the visual feature and the auditory pitch were varied randomly within each block. The visual feature and the auditory feature were collapsed across congruency to make a single variable: congruency. Congruency of a trial could be either congruent if the relevant visual and auditory features aligned, or incongruent if they did not. Congruent pairing of audio-visual features was defined in the three experiments as high elevation/high pitch and low elevation/low pitch; light/high pitch and dark/low pitch; and small/high pitch and large/low pitch (with the reverse pairings being incongruent).

### Stimuli and procedure

All experiments were competed on a Dell OptiPlex 7060 with an i5-8500 Intel 3.8Ghz core (6 CPUs), 16GB RAM, Microsoft Windows 10 Education 64-Bit (Version 10.0, Build 19043), and using an Acer KG271 LCD monitor (1,920 × 1,080 pixels). Auditory stimuli were presented using Audio-Technica ATH-M20x headphones. All measures of luminance and chromaticity were taken using a ColorCal Mk II Colorimeter. Participants were positioned approximately 70cms from the display and instructed to remain in this position for the duration of the experiment.

**Fig. 1:**
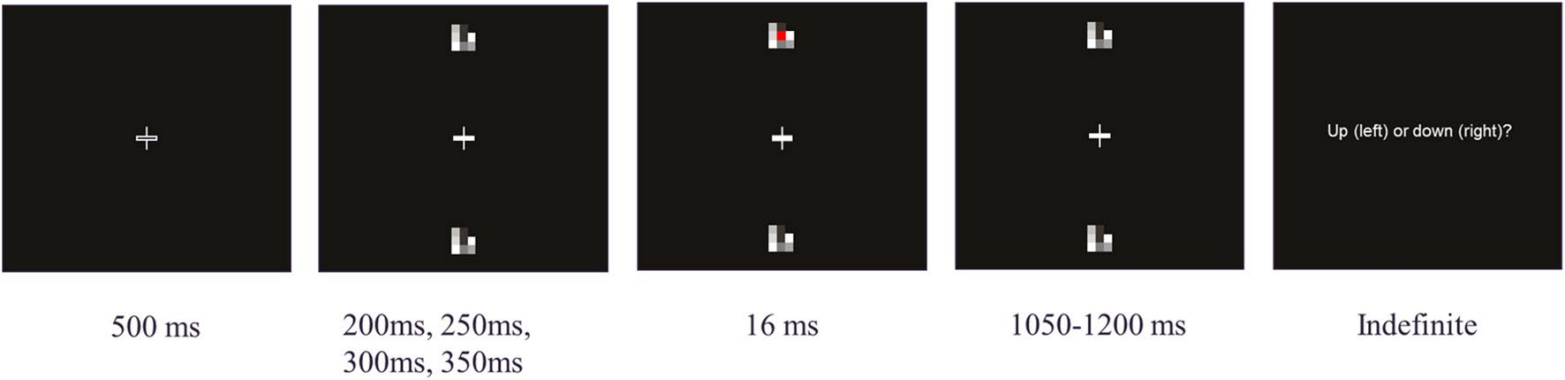
Read from left to right. Mock-up of an “up” trial from the elevation/pitch task

In all experiments, participants were presented with a black background and a white fixation cross (0.6 degrees diameter) in the centre of the screen. After 500 ms, participants were presented with two 3 × 3 arrays of small square elements, the centre square being offset vertically 6 degrees above and below fixation. In Experiment 3, this was changed to an array of 5 × 5, to account for variation of the stimulus size. Each element was 0.33 visual degrees in size and varied randomly in monochrome intensity every 16 ms. The luminance of the visual noise varied from 0.38 cd/m^2^ at the dimmest value to 212.6 cd/m^2^ at the brightest. After a randomly jittered duration (200 ms, 250 ms, 300 ms, 350 ms), the visual stimulus was presented over the centre square of one array (above or below), accompanied by the irrelevant auditory stimulus, each for 16 ms. The visual stimulus was a monochromatic square (0.33 degrees), either red (RGB: 255, 0, 0; CIE: x = 0.658, y = 0.318; luminance: 44.96 cd/m^2^) or blue (RGB: 0, 0, 255; CIE: x =165, y = 0.043; luminance: 9.18 cd/m^2^) depending on the colour condition. The auditory stimulus was a pure sinusoidal tone (750 Hz or 1500 Hz) and presented binaurally. In Experiment 1, the visual stimulus varied in elevation and pitch on a trial by trial basis, and participants used the left and right arrow keys to identify if the target was above or below fixation (left for above, right for below). Elevation continued to vary in Experiments 2 and 3 to prevent participants becoming attuned to the presentation location, but was not a factor in the analysis. In Experiment 2, the visual stimulus varied in lightness (dark red RGB: 125, 0, 0; CIE: x = 0.634, y = 0.311; luminance: 7.84 cd/m^2^; light red RGB: 255, 0, 0; CIE: x = 0.658, y = 0.318; luminance: 44.96 cd/m^2^; dark blue RGB: 0, 0, 100; CIE: x = 0.171, y = 0.05; luminance: 1.02 cd/m^2^; light blue RGB: 0, 0, 255; CIE: x = 165, y = 0.043; luminance: 9.18 cd/m^2^), and participants made keyboard responses to identify if the target was dark or light. In Experiment 3, the visual stimulus varied in size. To achieve this, the small visual stimulus covered only the central element of a 5 × 5 array, while the large visual stimulus covered a 3 × 3 area in the centre of the larger 5 × 5 array. To keep the difficulty of the size task in line with the other two, the targets were made partially transparent (red large: 70%, red small: 42%, blue large: 60%, blue small: 22%). As in the previous two experiments, participants made keyboard responses to discriminate the size of target as big or small. All experiments were completed twice, once for each colour in a random order. Figure 1 below shows the procedure of the elevation/pitch task as an example.

Before beginning the experiment, participants were asked to adjust the sound to a clear but comfortable level. They then completed a short practice (16 trials) to familiarize themselves with the experiment. The practice block used red visual stimuli. One practice task was completed for each experiment, immediately before beginning the relevant experiment. Each experiment consisted of 256 trials, 128 of each colour. The colour block order was counterbalanced, and within each, the relevant visual feature and irrelevant auditory feature varied randomly. Each block included four breaks, for a total of eight breaks for each experiment.

## Results

For all analyses, the discriminated visual feature and pitch variables were combined to categorize trials as ‘congruent’ or ‘incongruent.’ In the elevation/pitch experiment, high elevation paired with high pitch and low elevation with low pitch were combined into the congruent trials. For lightness/pitch, the congruent pairings were light/high pitch, and dark/low pitch. For size/pitch, the congruent pairings were small/high pitch, and large/low pitch. For each experiment, the reverse pairings were marked as ‘incongruent’ trials. The sensitivity (*d*′) to discriminating the visual feature was then determined for each participant in each experiment on an overall basis and separately for congruent audio-visual pairings and for incongruent audio-visual pairings.

The data were screened for overall *d*′s either above 3.5 or below 0.1. In addition, we screened for congruent and incongruent *d*′s above 3.5 or below −0.1. Participants who scored a *d*′ above 0.1 overall were included even if one of their congruency condition *d*′s was as low as −0.1, to allow for the possibility that they performed at chance in one condition but improved in the other (Zeljko et al., 2019). Participants with scores outside this range were removed from analysis. This was to prevent ceiling effects or guessing from influencing the analysis and allow for a repeated-measures analysis of variance (ANOVA). Scores of exactly 0 (random) have been included to allow for the possibility that a participant may be discriminating at chance for one condition, but better than chance for the other. This resulted in the removal of 10 individuals from the elevation/pitch experiment (all for ceiling effects), 11 from the lightness/pitch experiment (two for random responses, nine for noncompliance), and three from the size/pitch experiment (all for noncompliance). Removals in the elevation/pitch experiment were predominantly for *d*′s above 3.5 while removals in the other two were predominantly for noncompliance, as evidenced by large negative *d*′s, suggesting they were able to discriminate the targets, but still responded in an incorrect manner. The final participant numbers for each experiment were 24 for elevation/pitch, 19 for lightness/pitch, and 27 for size/pitch. Effect sizes are reported in the form of partial eta-squared for ANOVAs, and Cohen’s *d* for paired *t* tests. The pattern of results does not change if these participants are included.

### Elevation/pitch

A repeated-measures ANOVA was conducted to examine the influence of colour and elevation/pitch congruency on the sensitivity to elevation discrimination. This revealed a main effect of colour, *F*(1, 23) = 19.71, *p* < .001, η_p_^2^ = .462, no main effect of congruency *F*(1, 23) = 1.97, *p* = .174, and a significant Colour × Congruency interaction, *F*(1, 23) = 8.92, *p* = .007, η_p_^2^ = .280. Follow up paired comparisons showed that when the target was red, sensitivity was significantly greater on congruent trials than incongruent, *t*(23) = 3.52, *p* = .002, Cohen’s *d* = .719, but there was no sensitivity benefit of congruency when the target was blue, *t*(23) = −0.75, *p* = .458. The significant difference in the follow up paired comparison between congruent and incongruent trials when the target was red remains significant when a Bonferroni correction is applied for two comparisons.

### Lightness/pitch

A repeated-measures ANOVA was conducted to examine the influence of colour and lightness/pitch congruency on the sensitivity to lightness discrimination. This revealed no main effect of colour, *F*(1, 18) = 1.65, *p* = .215, a main effect of congruency *F*(1, 18) = 8.62, *p* = .009, η_p_^2^ = .063, and no Colour × Congruency interaction, *F*(1, 18) = 1.32, *p* = .265. Given that our interest was in comparing congruency effects within each colour condition, we conducted planned follow-up comparisons which revealed that when the target was red, sensitivity was significantly greater on congruent trials than incongruent, *t*(18) = 2.752, *p* = .013, Cohen’s *d* = .631. When the target was blue, sensitivity was not significantly greater on congruent trials than incongruent after we apply a Bonferroni correction, *t*(18) = 2.246, *p* = .037, Cohen’s *d* = .515. This could be argued as ‘marginally significant’, particularly in light of the comparably equal effect size to the congruency effect in the red condition, and the implications of this are considered in the discussion. It is also worth noting that Bonferroni corrections are a highly conservative method of controlling for family wise error rate. This method was preferred due to the mixed evidence of the S-cone methodology, and so the importance of rejecting Type I errors.

### Size/pitch

**Fig. 2:**
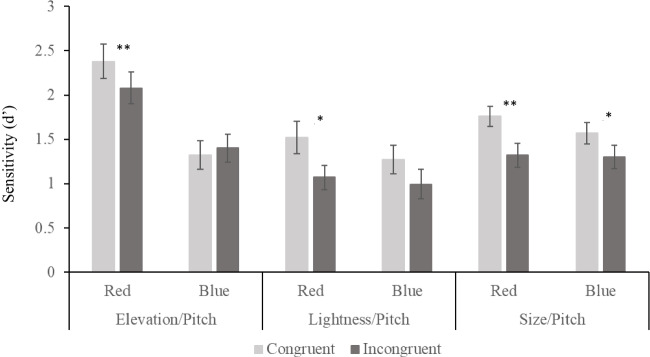
Discrimination sensitivity to the visual feature for each experiment, split by colour and congruency. Asterisks indicate significance of congruent versus incongruent *t* tests: *p < .05. **p < .01

A repeated-measures ANOVA was conducted to examine the influence of colour and size/pitch congruency on the sensitivity to size discrimination. There was no main effect of colour, *F*(1, 26) = 0.56, *p* = .461, a main effect of congruency, *F*(1, 26) = 11.802, *p* = .002, η_p_^2^ = .312, and no Colour × Congruency interaction, *F*(1, 26) = 1.605, *p* = .216. Again, we conducted planned follow-up comparisons to explore congruency within each colour condition. These found that when the target was red, sensitivity was significantly greater on congruent trials than incongruent, *t*(26) = 3.315, *p* = .003, Cohen’s *d* = .638. Similarly, when the target was blue, sensitivity was significantly greater on congruent trials than incongruent, *t*(26) = 2.48, *p* = .020, Cohen’s *d* = .477. This effect remains significant after a Bonferroni correction for two comparisons. The results of the follow-up comparisons for each experiment can be seen in Fig. 2.

Finally, we considered the possible effect of elevation/pitch congruency on the lightness/pitch and size/pitch experiments. Some research (e.g., Evans & Treisman, 2010) have found CMC effects on discriminations of CMC irrelevant features (e.g., elevation/pitch congruence affects the discrimination of some visual feature other than elevation). We considered it would be appropriate to check for, and eliminate this possibility, as elevation does vary throughout our lightness and size discrimination tasks. Due to elevation/pitch producing different congruent and incongruent *d*′s in the lightness/pitch and size/pitch task, different participants were excluded based on their *d*′, but based on the same criteria as used initially. This resulted in a removal of seven individuals from the lightness/pitch analysis and three from the size/pitch analysis. In the lightness/pitch task, we found no main effect of colour, *F*(1, 22) = 3.79, *p* = .064, no main effect of elevation/pitch congruency, *F*(1, 22) = 2.28, *p* = .145, and no Colour × Congruency interaction, *F*(1,b22) = .003, *p* = .957. Similarly, in the size/pitch task, we found no main effect of colour, *F*(1, 26) = 1.42, *p* = .243, no main effect of elevation/pitch congruency, *F*(1, 26) = .619, *p* = .438, and no Colour × Congruency interaction, *F*(1, 26) = .016, *p* = .899. These results are consistent with Zeljko et al. (2019) who found no effect of elevation/pitch congruency on lightness discriminations and no effect of lightness/pitch congruency on elevation discriminations. We conclude from this that our lightness/pitch and size/pitch tasks were not affected by the variation of elevation. Further to this point, we do not consider the variation between the luminance in the red and blue conditions to be a confound. Zeljko et al. (2019) finding no effect of lightness on elevation discriminations strongly suggests our Colour × Congruency interaction in the elevation/pitch task was unaffected by the differences in luminance.

## Discussion

Following the experimental design of Leo et al. (2008) and Bertini et al. (2008), we used variation in the colour of light to project our visual stimulus selectively to the SC in some conditions but not others. Specifically, red light was used for targets intended to reach the superior colliculus, while blue light was used to hide targets from the SC. We predicted that when the superior colliculus did not have access to the visual information, the relevant crossmodal congruency effect in each task would not manifest. In this way, we were able to examine how basic audio-visual crossmodal congruency effects manifest with or without superior colliculus involvement. As predicted, all three CMCs exhibited significant congruency effects for red stimuli. For blue stimuli, there was no congruency effect in the elevation/pitch task, a marginal effect in lightness/pitch task, and a significant congruency effect in size/pitch task. First, we look at each crossmodal correspondence in isolation and make a determination about the involvement of the superior colliculus. After this, we discuss the overall pattern of results and what they suggest for the processing of crossmodal correspondences generally.

Regarding the elevation/pitch CMC, it is apparent that the elevation discrimination sensitivity benefit conferred by congruent pairings of elevation/pitch does not manifest if the visual target does not project to the SC. The elevation/pitch correspondence is one of the best studied and replicated effects in crossmodal correspondence literature (Spence, 2011), and its absence in the blue condition is notable. It also seems appropriate here to address the main effect of colour which did not appear in the other two conditions. Although in the red condition it was significantly easier to discriminate elevation than the blue condition, neither were close to ceiling or floor effects. We can see in both the other two experiments, as well as in the results of Zeljko et al. (2019), that the range of *d*′s in the blue condition are well within a range that is appropriate for congruency effects to manifest. Unlike the other blue experiments with a similar range of *d*′s, however, there was no congruency effect in the elevation/pitch experiment. Furthermore, we are not doing any specific analysis by colour this main effect might influence. Our analysis of congruency is investigating the pairing of the visual feature (elevation/lightness/size), with the auditory feature, within each colour condition. One might propose that there is an effect of visual stimuli salience on congruency effects manifesting, such that it only manifests in the higher salience red condition, but again, this conflicts with the findings of the other two experiments where the d’s in the blue condition of elevation/pitch was not out of line with the blue condition in the other two experiments.

Turning to the lightness/pitch experiment, we suggest the results likely indicate that the SC is not required for the lightness/pitch CMC. Although the blue condition did not produce significant congruency effects, this is only due to the Bonferroni correction. This experiment included a considerably reduced number of participants in the final analysis, due to the difficult nature of detecting shades of blue light. At 19 participants, it was possibly underpowered, and we believe the congruency effect in the blue condition may be significant with greater participant numbers.

Finally, the size/pitch experiment provides good evidence that the size/pitch CMC does not require SC involvement for congruency effects to manifest. Considering the low-level sensory role of the SC, this was in line with our expectations. Size as a visual feature is more complex than lightness or elevation and would require multiple neurons encoding together across space. It is possible the superior colliculus is unable to encode this more complex visual attribute and so the size/pitch correspondence must be occurring later in the visual pathway. Evidence from fMRI studies such as Tanaka and Fujita (2015) and Sperandio et al. (2012) suggest visual size is determined by processing in V1 and V4. It would not be possible for sensory regions earlier than this to make use of size information in neural coactivation.

Considering the experiments together generates suggestions of general findings about crossmodal correspondences from the pattern of results. The three experiments represent three increasing levels of complexity in their visual feature. Elevation is the simplest, because both the superior colliculus and the visual cortex are retinotopic. A neuron will code for elevation inherently by activation. Lightness is slightly more complex because it is encoded by neuronal firing rates. Last is size, which as described earlier will require multiple neurons to work in unison to encode this feature. By comparing between these experiments, we can make inferences about both how and where CMCs overall are processed in the brain.

The question of the involvement of the superior colliculus in CMCs can be split into three concepts. First: the superior colliculus is directly involved in CMC processing, either providing congruency benefits or encoding and transmitting congruency information to higher processing areas (e.g., sensory cortex) that then provide the benefit. Second: the superior colliculus is indirectly involved in CMC processing, not encoding or recognizing congruent crossmodal features, but rather, providing the integration of the crossmodal signals that is necessary for CMC processing later in the perceptual system. Third: the superior colliculus is entirely uninvolved in CMC processing.

Addressing the first possibility of direct SC involvement, our results are plausibly consistent with this interpretation. The SC is organized retinotopically, and thus can detect visual elevation (Chandrasekaran et al., 2005; Katyal et al., 2010). We also have evidence that pitch information is processed in the SC. First, the inferior colliculus is able to distinguish both pitch and rhythm, and feeds information onto the superior colliculus (Driscoll & Tadi, 2022). Second, this has been demonstrated in pigtail macaques, who show differential activation of the SC to high- and low-pitched vocalizations—high pitched indicating threatening behaviour (Desjardin et al., 2013). With this information, the elevation/pitch correspondence may be occurring in the SC itself. Conflicting with this, however, is the finding that lightness/pitch does not appear affected by superior colliculus involvement. To resolve this requires some nuance. The superior colliculus may be able to encode for lightness. This does not necessarily mean the neurones of the SC are considering this information when processing stimuli. For its role in spatiotemporal integration, the feature of lightness is irrelevant. On the other hand, elevation is a spatial construct, and we can reliably say that the neurones of the SC will consider elevation in space when performing spatiotemporal integration. Evidence for this is recognition that an elevated visual target spatially coincides with an elevated auditory target. Combined with the evidence that high pitched stimuli tend to come from above in the environment (Parise et al., 2014), we can imagine how the brain would develop to fine tune its spatiotemporal integration for statistical regularities of spatial and auditory features.

This possibility has interesting implications for our categorization of CMCs as a whole. If elevation/pitch is processed in the superior colliculus, while lightness/pitch and size/pitch are not, it calls into question why these effects are grouped together at all. Although we can observe superficially similar effects from CMCs, such as in Zeljko et al. (2019), the brain is performing these pairings in different ways. Adding further to this point, the effects of Zeljko et al. showed specificity to the feature being discriminated. The elevation/pitch correspondence did not affect lightness discrimination, and vice versa. An interpretation in line with this proposal is that these effects are dissociable. CMC processing is not clustered into a singular ‘CMC region’ or module, but distributed throughout the sensory system, and reflect different regions independently becoming attuned to the statistics of the environment. This line of thought could be verified using fMRI. If elevation/pitch is different from lightness/pitch and size/pitch, it might be possible to further differentiate lightness/pitch from size/pitch correspondence. Lightness/pitch might occur at V1, while size might occur later at V4, and this would be reflected in the BOLD signal associated with each correspondence.

The second possibility, that the superior colliculus is involved only in performing spatiotemporal integration which is required later in the perceptual system for CMC congruency effects is unlikely. All three experiments make use of spatiotemporal integration, by presenting a visual stimulus that temporally coincides with the auditory stimulus. Why would only elevation/pitch require spatiotemporal integration for it to later manifest congruency effects, if lightness/pitch and size/pitch apparently do not? If anything, we could expect the opposite pattern if this were true with the more basic correspondence being more robust, and the more complex correspondence requiring prerequisite information.

The final possibility is the most unlikely, if we accept that the use of red and blue light has sufficiently modulated superior colliculus involvement. Following the design of Leo et al. (2008) and Bertini et al. (2008), we believe there is good evidence that this method is effective at selectively inhibiting superior colliculus activity during a trial. Short wavelength light is processed by S cones in the retinal, which project to koniocellular cells in the LGN, which do not feed into the superior colliculus. Visual information is projected to the superior colliculus from the magnocellular pathway. In addition, we control for cortico-tectal projects from V1 to the SC using forwards and backwards luminance noise as such a pathway would only project luminance information. Taking this into account, the evidence suggests that superior colliculus involvement is necessary for elevation/pitch correspondence congruency effects to manifest in elevation discrimination. This is incompatible with any theories which argue that the superior colliculus is entirely uninvolved in CMC processing.

If we do not accept that the use of red and blue light is capable of modulating SC activity based on animal studies and propose that the SC is receiving inputs during both red and blue trials, the question remains of how this specific pattern of data has occurred. Consistent congruency effects across all conditions might support this claim. In our study, the CMC most likely to rely on SC activity (elevation/pitch), is also the only CMC to show colour related interactions, while the other CMCs, especially size/pitch (likely too complex for SC processing) does not. This strongly supports the notion that our S-cone method was successful.

There does exist an alternative account for this data, which still supports SC CMC processing without relying on S-cone isolation. As observed in animal models, there is a noticeable delay in the arrival of chromatic information to the SC compared with luminance information. This difference has been described as anywhere between 15 and 20 ms (White et al., 2009) to 20–40 ms specifically for S-cone information (Hall & Colby, 2014). Bottom-up multisensory integration, performed by the SC, relies on overlap of multisensory signals in space and time. As a result of the disparity in the processing time of signals from different modalities, the SC does allow some leeway in temporal overlap as requiring an exact coincidence of signals would preclude most visuotactile and audiovisual binding. Meredith et al. (1987) found that this window of binding depended on the length of the unisensory stimuli in cat models. As the length of the unisensory stimuli increased, so too did the window in which cells with overlapping receptive fields from different modalities were able to combine information. Our use of extremely short 16ms stimuli, combined with the slowed transmission of S-cone activity, may have prevented crossmodal binding in the superior colliculus due to asymmetry of signal arrival in the SC. This possibility would still support our claim for elevation/pitch crossmodal correspondence in the SC, but would account for animal models suggesting that S-cone activity may have been present in the SC during blue trials.

Overall, these results may suggest that the superior colliculus is required in CMC processing, at least in at least one pairing. These findings provide a unique insight into the manner in which CMC processing occurs in the brain. Our findings suggest that the superior colliculus is required for the elevation/pitch correspondence and suggests that elevation/pitch is different in nature from other correspondences. This calls into question the categorization of CMCs as a whole, as their superficial behavioural similarities become less relevant with a better understanding of the neural processes behind them. We believe this question of differential neuronal CMC processing is ripe for neuroimaging follow-ups, as well as verification of these findings in animal models. Improvements in our neural model of CMC processing may eventually answer long-held questions regarding the influence of environmental statistics or innate developmental factors in crossmodal correspondence.

## Declarations

### Open practices statement

All data and materials have been made available online on Open science framework at: https://osf.io/t73dh/.
